# Exploring the determinants of non-suicidal self-injury among university students in Jordan: a cross-sectional study

**DOI:** 10.3389/fpsyt.2025.1751707

**Published:** 2026-01-23

**Authors:** Hana Taha, Omar AlHaddad, Suhib Awamleh, Abdallah Shatnawi, Habib Yazan Belkhiria, Yahia Jarosheh, Taher Alhawamdeh, Ayman M. Wahbeh, Abdulla Al-Ani, Linus Jönsson

**Affiliations:** 1 Department of Family and Community Medicine, School of Medicine, The University of Jordan, Amman, Jordan; 2 Department of Neurobiology, Care Sciences and Society, Karolinska Institutet, Solna, Sweden; 3 Primary Healthcare Department, King Khalid Hospital, Kharj, Saudi Arabia; 4 Department of Internal Medicine, School of Medicine, The University of Jordan, Amman, Jordan; 5 Division Vascular and Interventional Radiology, Boston Children’s Hospital, Boston, MA, United States

**Keywords:** anxiety, depression, Jordan, non-suicidal self-injury, university students

## Abstract

**Background:**

Deliberate bodily harm without suicidal intent has become a growing concern among young adults and adolescents. This study aims to explore the determinants of non-suicidal self-injury (NSSI) and its correlation with psychological distress and mobile phone addiction (MPA) among university students in Jordan.

**Methods:**

A quantitative cross-sectional study targeting university students in Jordan. The data was collected from 394 students using a semi-structured online questionnaire that included socio-demographic characteristics; Self-Harm Inventory scale (SHI); Patient Health Questionnaire-9 (PHQ-9); General Anxiety Disorder-7 (GAD-7); and Smartphone Addiction Scale-Short Version (SAS-SV). SPSS 30 was used for descriptive and multivariate analysis. Spearman’s correlation, Chi-square, Kruskal—Wallis and Mann—Whitney U were applied. Binary logistic regression was conducted to identify the predictors of significant NSSI.

**Results:**

Prevalence of significant self-harm (threshold of SHI ≥5) was 20.56%. Higher levels of anxiety and depression were strongly associated with increased NSSI severity (p <.001). Depression was a strong predictor of significant self-harm (p <.001, OR = 1.21). Participants who reported experiencing negative or traumatic events during childhood, or having a family member with a psychological disorder, were significantly more likely to fall into the higher-risk NSSI category (p <.001).

**Conclusion:**

This study demonstrated alarming levels of self-harm behaviors among university students in Jordan as measured by SHI. NSSI was strongly associated with psychological distress and dysfunctional family environment, which may provide insight into its etiology and has implications for early identification and intervention.

## Background

Non-suicidal self-injury (NSSI) is defined as direct and deliberate bodily harm without suicidal intent (1). NSSI is a persistent mental health concern, particularly among adolescents and young adults. Based on meta-analysis evidence from nonclinical community samples, the estimated overall NSSI prevalence was 17.2% among adolescents, 13.4% among young adults (2). The most common forms of NSSI include cutting and carving the skin, and these behaviors show consistent patterns globally. Meta-analytic evidence indicates that NSSI most often serves intrapersonal functions (e.g., reducing negative states or producing relief), but it may also fulfill interpersonal functions, such as eliciting social support or avoiding social demands (3). Evidence indicates that NSSI is closely associated with mental health challenges, particularly social anxiety (4).

NSSI has emerged as an increasing concern among university students. A study from the West and Northwest provinces of Iran reported a notably high lifetime prevalence of 29.3% in this population (5). Beyond psychological correlations, behavioral factors such as mobile phone addiction have also been shown to increase the odds of NSSI among college students (6, 7). Non-suicidal self-injury (NSSI) has been documented at varying rates across university settings, highlighting its significance as a mental health concern. Research among large student samples shows that prevalence rates differ widely, ranging from 7% lifetime prevalence in a U.S. college cohort, with 2% reporting NSSI in the past year (8), to 17.7% among 20,842 first-year students from 24 international colleges (9). In addition, a meta-analysis of 62 studies that included over 264,638 adolescents identified a lifetime prevalence of 22.0% and a 12-month prevalence of 23.2%, with repetitive NSSI (20.3%) being more common than episodic NSSI (8.3%), and multiple-method self-injury (16.0%) occurring more frequently than single-method self-injury (11.1%) (10). Across these studies, females reported higher rates of engagement in NSSI than males. Having siblings, living to a single-parent family and smoking were linked to higher prevalence of NSSI (10).

Despite the increasing recognition of its clinical significance, important gaps remain in understanding the determinants of NSSI among university students. NSSI has been strongly associated with psychological distress, particularly depression and anxiety. Research among Jordanian university students identified social anxiety as the most significant predictor of deliberate self-harm, with depression and general anxiety having lower associations (4). Similarly, studies among medical students revealed high rates of depression (58.6%) and suicidal ideation (27.4%), with family problems, drug use, and relationship status acting as important contributing factors (11). Large-scale international research further showed that NSSI predicts later mental health disorders, with one study among 20,842 first-year university students reporting that engagement in NSSI increased the odds of developing a mental disorder by nearly twofold (OR = 1.8) (9).

Beyond mental health symptoms, behavioral patterns have also emerged as significant correlates of NSSI. Problematic technology use has been increasingly linked to self-injurious behaviors. A study of 18,723 college students found that mobile phone addiction (MPA) was reported by 29.7% of participants and was significantly associated with NSSI, which had a prevalence of 6.5% in the sample. Students with MPA were more than twice as likely to engage in self-injury (OR = 2.12), with the association being stronger among females (OR = 1.31) (7). Similarly, research on internet addiction and self-concealment revealed positive correlations with NSSI among university students, suggesting that maladaptive coping strategies and difficulties with emotional expression may heighten vulnerability (6). These findings highlight the importance of considering technology use as a modern behavioral factor that intersects with student mental health.

Based on the latest global statistics, suicide was the third leading cause of mortality among those aged 15–29 years, and 73% of all the suicides occurred in low- and middle-income countries (12). During the Covid-19 Pandemic, previous history of depression, self-harm, and suicidal attempts were risk factors for relapsing depression and suicidality (13). Although NSSI is conceptually distinct from suicidality, evidence shows that these behaviors often overlap, which amplifies their clinical significance (14). Studies conducted in Indonesia showed that the most significant predisposing factors of NSSI behaviors among youth were introverted personality, environment that nurture NSSI behaviors and maladaptation (15, 16). While bullying and deep disappointment were the precipitating factors related to the dynamics of NSSI behaviors (16). Based on integrated behavioral models, NSSI is the entry step that enables teens and young adults’ potential for suicide (14). In young people with a previous history of NSSI, nearly 70% have attempted suicide at least once and 55% multiple times (17, 18).

A cross-national study involving 8,417 students from 12 Muslim-majority countries reported that 22.1% experienced suicidal ideation, while 8.6% had attempted suicide, with women and younger students at greater risk (19). Despite growing international evidence, there remains limited research exploring the determinants of NSSI among young people in the Jordanian context, particularly in relation to psychological and behavioral factors such as mobile phone use. Addressing this gap, the purpose of this study is to investigate and understand the determinants that contribute to NSSI among university students in Jordan.

## Methods

### Study design

This is a quantitative cross-sectional study that will give a snapshot of NSSI and its determinants among a nonclinical sample of university students in Jordan.

### Study setting

This study was conducted in 16 universities in Jordan (6 public and 10 private) to capture diversity and gain deeper insights into potential patterns contributing to NSSI and to facilitate broad data collection. The data was collected during the second academic semester between February and June 2025. The timing was selected to align with the availability of the targeted population in the academic setting.

### Sampling strategy and participants

This study employed a convenience sampling method to recruit students from universities across Jordan, where participants were approached and recruited based on their availability. Students from universities across Jordan were the target population, as NSSI had not been specifically or thoroughly studied in this context. Eligibility criteria included all the students from universities across Jordan who consented to participate in the study. Single population proportion formula was used to calculate the sample size. n = z^2^pq/d^2^.

Where, n = sample size, z = level of confidence according to the standard normal distribution (for a level of confidence of 95%, z = 1.96), p = estimated proportion of the population that presents the characteristic (Here we use p = 0.5), d = tolerated margin of error (for example we want to know the real proportion within 5%). Thus, placing all values, Sample size = (1.96)^2^ (0.5) (0.5)/(0.05)^2^ = 384.16. Thus, approximately a total of 394 had been included in the final analysis, providing a diverse representation of the university students population from various socio-economic and demographic backgrounds.

### Recruitment and participation

The data were collected using an online questionnaire (20). Participants were recruited through announcements shared in social media groups and platforms that are used exclusively by students at the participating universities, and through a quick response “QR” code. The invitation to participate in answering the study tool included a summary of the research objectives, methods, clear statement of the voluntary nature of participation, and a written consent icon that must be checked before completing the questionnaire. Due to the use of open online recruitment via student social media groups and QR codes, the total number of students who were exposed to the survey invitation could not be precisely determined. As such, a formal response rate could not be calculated. However, online data collection was the most appropriate for this research to safeguard privacy and ensure anonymity because NSSI is a sensitive subject that is stigmatized and considered a taboo (21).

### Measurement tool

We used a semi-structured questionnaire that included socio-demographic measurable, mobile phone addiction, depression, anxiety, and non-suicidal self-injury. Non-suicidal self-injury was measured using the Self-Harm Inventory scale (SHI). SHI is a 22-item binary-response questionnaire designed to evaluate individuals’ history of intentional self-harm. Total scores reflect the number of endorsed behaviors, with a maximum count of 22 (22). Depression was evaluated using the Patient Health Questionnaire-9 (PHQ-9) (23), anxiety was evaluated using General Anxiety Disorder-7 (GAD-7) (24). Mobile phone addiction levels were measured using the Smartphone Addiction Scale-Short Version (SAS-SV) (25).

### Ethical considerations

Ethical approval for this study was obtained from the Ethics Committee of the University of Jordan Hospital. Participants were informed about the study objectives, and their voluntary participation was ensured. Anonymity was maintained, as no identifying information was collected, ensuring confidentiality throughout the study.

### Data analysis

All analyses were conducted using IBM SPSS Statistics (Version 27). Normality of the main study variables was assessed using the Shapiro–Wilk test, histograms, and Q–Q plots. As most variables deviated from normal distribution including Self-Harm Inventory (SHI) scores—non-parametric tests were applied where appropriate.

The internal consistency of all psychometric instruments was examined using Cronbach’s alpha, with all scales demonstrating excellent reliability: Smartphone Addiction Scale–Short Version (SAS-SV, α = .917), Generalized Anxiety Disorder Scale (GAD-7, α = .921), Patient Health Questionnaire (PHQ-9, α = .897), and Self-Harm Inventory (SHI, α = .906).

To examine group differences in SHI categories, Chi-square tests were used for categorical variables where assumptions were met, including some with multiple categories. However, for variables that violated Chi-square assumptions due to small, expected cell sizes, the Kruskal–Wallis test was employed as a non-parametric alternative for multi-category variables, and for the GPA, Mann–Whitney U test was used to compare GPA scores between two independent groups based on their SHI category (significant vs. non-significant SHI). This allowed for valid comparisons without the constraints of distributional assumptions or equal group sizes.

The SHI assesses a broad spectrum of self-destructive behaviors, including cognitive, relational, and behavioral items, some of which extend beyond Diagnostic Manual of Mental Disorders (DSM-5) (26)and International Classification of Diseases (ICD-10-CM) definitions of NSSI (27, 28). Accordingly, endorsement of one or more SHI items was not interpreted as clinical NSSI prevalence. Instead, SHI scores were used dimensionally, and a cut-off of ≥ 5 was applied to identify significant self-harm, consistent with prior literature (22, 29). The SHI scale was used in this study as a screening tool to assess the lifetime prevalence of the 22 self-harm behaviors listed in the instrument (22, 29) including items referring to suicidal behaviors (e.g., suicide attempts, overdosing), which reflect the empirically documented overlap between self-harm and suicidality in university (30).

Variable categorization was conducted as follows: SHI scores were dichotomized into non-significant self-harm (0–4) and significant self-harm (5–22) (22, 29). For SAS-SV, males were categorized as addicted with scores ≥31, and females with scores ≥33 (25). GAD-7 scores were classified into minimal (0–4), mild (5–9), moderate (10–14), and severe anxiety (15–21) (24). PHQ-9 scores were grouped into minimal (0–4), mild (5–9), moderate (10–14), moderately severe (15–19), and severe depression (20–27) (23).

To identify multivariate predictors of significant non-suicidal self-injury, we conducted a stepwise binary logistic regression. The dependent variable was dichotomized into “non-significant” and “significant” self-harm based on SHI scores. An *a priori* set of candidate predictors was initially considered, including: PHQ-9, GAD-7, and SAS-SV total scores; gender; age; birth order; academic major; living situation; monthly family income; university; history of negative or traumatic childhood experiences; and having a family member with a psychological disorder. The stepwise selection process and sequence of variable entries are detailed in **Table A1** in **Appendix A**. Following the stepwise selection procedure, the final model retained PHQ-9 score, SAS-SV score, gender, history of negative or traumatic childhood experiences, and having a family member with a psychological disorder as significant predictors.

Model adequacy and predictive performance were evaluated using several diagnostic procedures. Overall model fit was examined using the Omnibus Test of Model Coefficients to determine whether the set of predictors significantly improved prediction over the null model. The Hosmer–Lemeshow goodness-of-fit test was used to assess calibration between observed and predicted probabilities. The model’s explanatory power was quantified using Nagelkerke’s R² (31), and discriminative ability was evaluated through the Receiver Operating Characteristic (ROC) curve and associated classification accuracy indices. Multicollinearity was assessed using variance inflation factors (VIF) and tolerance values derived from linear regression diagnostics, with all predictors meeting accepted thresholds indicating no multicollinearity concerns.

## Results

### Study sample characteristics

In the 394 sample we included, more than half were females (53.6%). Most participants were between 18 and 24 years old (95.7%), with a mean GPA of 3.16. Furthermore, most participants were from the University of Jordan (53.8%), with the most common field of study being the medical field. Approximately 31% of participants were in their third year of study. The most reported income bracket was 300–800 JODs, with 34.5% of participants. Furthermore, the majority of participants (83.5%) reported living with their families. More than half of the participants (52.8%) reported being exposed to negative or traumatizing experiences while growing up. Most (83.5%) reported no history of mental illnesses in their family. The mean score for SHI was 2.83 out of 22 as showcased in **Table 1**.

**Table 1:** Baseline characteristics.

Variable		Category	N	%
Gender		Female	211	53.6%
		Male	183	46.4%
Age		18-24	377	95.7%
		25-31	13	3.3%
		32 and above	4	1.1%
Birth order		Youngest	80	20.3%
		Middle	183	46.4%
		Oldest	122	31.0%
		Only child	9	2.3%
GPA	N = 365 (29 missing)	Mean (± SD)	3.16	± 0.493
University		University of Jordan	212	53.80%
		Yarmouk University	70	17.80%
		Applied Science Private University	29	7.40%
		Jordan University of Science and Technology	22	5.60%
		Other public universities	31	7.90%
		Other private universities	28	7.10%
Major		Applied sciences	43	10.90%
		Arts	11	2.80%
		Business	19	4.80%
		Engineering	68	17.30%
		Humanities	48	12.20%
		Information technology	23	5.80%
		Law	13	3.30%
		Medical field	169	42.90%
Current semester credit hours	N = 390 (4 missing)	Mean (± SD)	16.27	± 4.057
Living situation		Living with colleagues	25	6.30%
		Living with family	329	83.50%
		Living alone	40	10.20%
Monthly income		<300	41	10.40%
		300–800	136	34.50%
		801–1200	102	25.90%
		1201–1700	41	10.40%
		1701–2200	20	5.10%
		>2200	54	13.70%
Year of study		1	66	16.80%
		2	76	19.30%
		3	120	30.50%
		4	76	19.30%
		5	36	9.10%
		6	12	3.00%
		Missing	8	2.00%
Did you lose someone you love in the last year		No	245	62.20%
		Yes	149	37.80%
any negative/traumatizing experience growing up		No	186	47.2%
		Yes	208	52.8%
Does anyone in your family suffer from a psychological disorder?		No	329	83.5%
		Yes	65	16.5%
SAS-SV		Mean score (± SD)	35.0711/60	± 11.89821
GAD-7		Mean score (± SD)	8.9848/21	± 6.09424
PHQ-9		Mean score (± SD)	11.3553/27	± 6.91502
SHI		Mean score (± SD)	2.8325/22	± 4.1575

### Group differences in NSSI severity based on psychological and demographic factors

The Chi-square analysis revealed several significant associations between psychological and psychosocial variables and the severity of self-harm behavior. Higher levels of anxiety and depression were strongly associated with increased NSSI severity. Specifically, participants with severe anxiety or depression were significantly more likely to report higher levels of self-harm, compared to those with minimal or mild symptoms (p <.001 for both GAD-7 and PHQ-9 categories).

Similarly, individuals who reported experiencing negative or traumatic events during childhood, or having a family member with a psychological disorder, were significantly more likely to fall into the higher-risk NSSI category (p <.001). In contrast, no significant association was found between smartphone addiction status and NSSI severity (p = .430), nor between gender or living situation and NSSI category as presented in **Table 2**.

**Table 2:** Chi-square test for group differences in NSSI.

Variable	Category	Non-significant level of Self-Harm (N)	Significant level of Self-Harm (N)	Chi-square	P-value
Gender	Female	171	40	0.713	0.398
	Male	142	41		
GAD-7 categories	Minimal anxiety	105	6	56.943	**<0.001**
	Mild anxiety	99	12		
	Moderate anxiety	70	29		
	Severe anxiety	39	34		
PHQ-9 categories	Minimal	62	2	77.658	**<0.001**
	Mild	105	11		
	Moderate	82	13		
	Moderately severe	41	25		
	Severe	23	30		
SAS-SV categories	Not addicted	135	31	0.623	0.43
	Addicted	178	50		
Did you lose someone you love in the last year	No	202	43	3.588	0.058
	Yes	111	38		
Any negative/traumatizing experience growing up	No	167	19	23.08	**<0.001**
	Yes	146	62		
Does anyone in your family suffer from a psychological disorder?	No	276	53	24.169	**<0.001**
	Yes	37	28		
Living situation	Living with colleagues	18	7	1.577	0.455
	Living with family	265	64		
	Living alone	30	10		

Bold values refers to significant p values.

### Item-level frequencies on the self-harm inventory

The categorization of SHI scores revealed that 80 students (20.56%) fell into the significant self-harm behavior category by scoring between 5 and 22. Among the study sample, 249 students (63.2%) reported lifetime prevalence of at least one self-harm behavior, while 36.8% scored zero on the Self-Harm Inventory (SHI), indicating no history of such behavior. To provide a more detailed understanding of the self-injurious behaviors reported in the sample, item-level frequencies from the Self-Harm Inventory (SHI) are presented in **Table 3**.

**Table 3:** SHI item-level frequencies.

Question	N (Yes)	% of total students with one item SHI (n = 249)	N (significant SHI group)	% of total students with significant SHI (n = 81)
1. Have you ever tortured yourself with self-defeating thoughts?	142	57.00%	67	82.70%
2. Engaged in emotionally abusive relationships?	115	46.20%	57	70.40%
3. Hit yourself?	82	32.90%	58	71.60%
4. Banged your head on purpose?	77	30.90%	48	59.30%
5. Driven recklessly on purpose?	67	26.90%	36	44.40%
6. Distanced yourself from God as punishment?	62	24.90%	38	46.90%
7. Scratched yourself on purpose?	59	23.70%	48	59.30%
8. Starved yourself to hurt yourself?	57	22.90%	43	53.10%
9. Made medical situations worse on purpose (e.g., skipped medication)?	55	22.10%	42	51.90%
10. Cut yourself on purpose?	50	20.10%	40	49.40%
11. Exercised an injury on purpose?	48	19.30%	43	53.10%
12. Scratched your wounds and prevented their healing?	40	16.10%	48	59.30%
13. Set yourself up in a relationship to be rejected?	39	15.70%	34	42.00%
14. Attempted suicide?	37	14.90%	32	39.50%
15. Abused prescription medication?	29	11.60%	57	70.40%
16. Burned yourself on purpose?	27	10.80%	24	29.60%
17. Been promiscuous (i.e., had many sexual partners)?	27	10.80%	21	25.90%
18. Engaged in sexually abusive relationships?	24	9.60%	18	22.20%
19. Overdosed medications?	20	8.00%	17	21.00%
20. Lost a job on purpose?	20	8.00%	12	14.80%
21. Did you abuse laxatives to hurt yourself?	20	8.00%	18	22.20%
22. Abused alcohol?	19	7.60%	19	23.50%

The most frequently endorsed behavior was *“torturing oneself with self-defeating thoughts”* (57.0%), followed by *“engaging in emotionally abusive relationships”* (46.2%), *“hitting oneself”* (32.9%), and *“banging one’s head on purpose”* (30.9%). Less frequently reported behaviors included *“overdosing”* (8.0%), *“losing a job on purpose”* (8.0%), and *“abusing laxatives”* (8.0%).

### Group differences in NSSI severity

A Mann–Whitney U test revealed no significant difference in GPA scores between the “non-significant SHI” group (Mean Rank = 184.21) and the “significant SHI” group (Mean Rank = 178.32), U = 10524.00, Z = -0.431, p = 0.666 (two-tailed). A series of Kruskal–Wallis tests were conducted to assess whether the severity of non-suicidal self-injury (NSSI) differed significantly across demographic groups (**Table 4**). A statistically significant difference was found based on year of study (H = 15.38, p = .009), with second-year students demonstrating the highest mean rank (221.97), followed by third-year students (208.16).

**Table 4:** Kruskal—Wallis tests.

Variable	Category	Mean rank	Kruskal—Wallis H	P value
Birth order	Only child	258.06	3.664	0.3
	Youngest	200.45		
	Middle	190.49		
	Oldest	201.61		
Major	Applied sciences	187.35	13.67	0.135
	Arts	277.64		
	Business	178.92		
	Engineering	197.65		
	Geography	237		
	Humanities	207.78		
	IT	198.13		
	Law	252.58		
	Medical field	188.28		
	Sports	309.75		
Monthly income	<300	197.67	1.457	0.918
	>2200	198.07		
	1201–1700	187.32		
	1701–2200	188.55		
	300–800	194.06		
	801–1200	207.56		
University	University of Jordan	185.58	12.43	0.029
	Yarmouk University	214.80		
	Applied Science Private University	189.79		
	Jordan University of Science and Technology	209.45		
	Other public universities	226.00		
	Other private universities	200.26		
Year of study	1	178.31	15.38	0.009
	2	221.97		
	3	208.16		
	4	176.27		
	5	162.63		
	6	151.92		
Age	18–24	196.25	6.17	0.187
	25–31	255.23		
	32 and above	146		

In contrast, fifth- and sixth-year students reported the lowest mean ranks (162.63 and 151.92, respectively), suggesting lower NSSI severity in later academic years. No significant differences in NSSI severity were found by birth order (H = 3.664, p = .300), study major (H = 13.67, p = .135), monthly income (H = 1.457, p = .918), or age group (H = 6.17, p = .187). Although some descriptive variation in mean ranks was observed, such as higher ranks among sports majors (309.75) and only children (258.06), these differences did not reach statistical significance.

### Multivariate analysis

Model fit was assessed using the Omnibus Test of Model Coefficients (χ²(5) = 104.134, p <.001), and the Hosmer–Lemeshow test confirmed good fit (χ²(8) = 4.555, p = .804). The final model explained 36.5% of the variance in self-harm severity (Nagelkerke R² = .365), with a classification accuracy evaluated using the Receiver Operating Characteristic (ROC) curve (32). Multicollinearity was assessed using linear regression diagnostics; all predictors demonstrated acceptable levels (VIF < 3, Tolerance > 0.2), indicating no concerns regarding multicollinearity.

**Table 5** presents the odds ratios (OR), and 95% confidence intervals for each significant predictor. Depression symptoms (PHQ-9) were a strong predictor of significant self-harm (p <.001, OR = 1.209), with each unit increase in PHQ-9 score associated with a 20.9% increase in the odds of falling into the high-risk group. Conversely, higher SAS-SV scores were associated with a small but significant decrease in odds of significant NSSI (p = .028, OR = 0.97).

**Table 5:** Multivariate analysis for the predictors of NSSI levels.

Predictors of NSSI levels	Category	Sig.	OR	95% C.I. of OR	
SAS-SV	–	0.028	0.97	0.944	0.997
PHQ-9	–	<0.001	1.209	1.147	1.274
Gender (Reference: Female)	Male	0.016	2.101	1.151	3.836
Any negative/traumatizing experience growing up (Reference: No)	Yes	0.014	2.261	1.178	4.342
Does anyone in your family suffer from a psychological disorder? (Reference: No)	Yes	0.003	2.806	1.433	5.496

Male gender was also significantly associated with higher odds of significant NSSI (p = .016, OR = 2.101). Additionally, students with a history of negative or traumatic childhood experiences (p = .014, OR = 2.261), and those with a family member suffering from a psychological disorder (p =.003, OR = 2.806), had significantly increased odds of being in the high-risk self-harm group. These findings indicate that while psychological symptomatology plays a substantial role, background experiences and psychosocial context also contribute meaningfully to self-harm severity.

**Figure 1:**
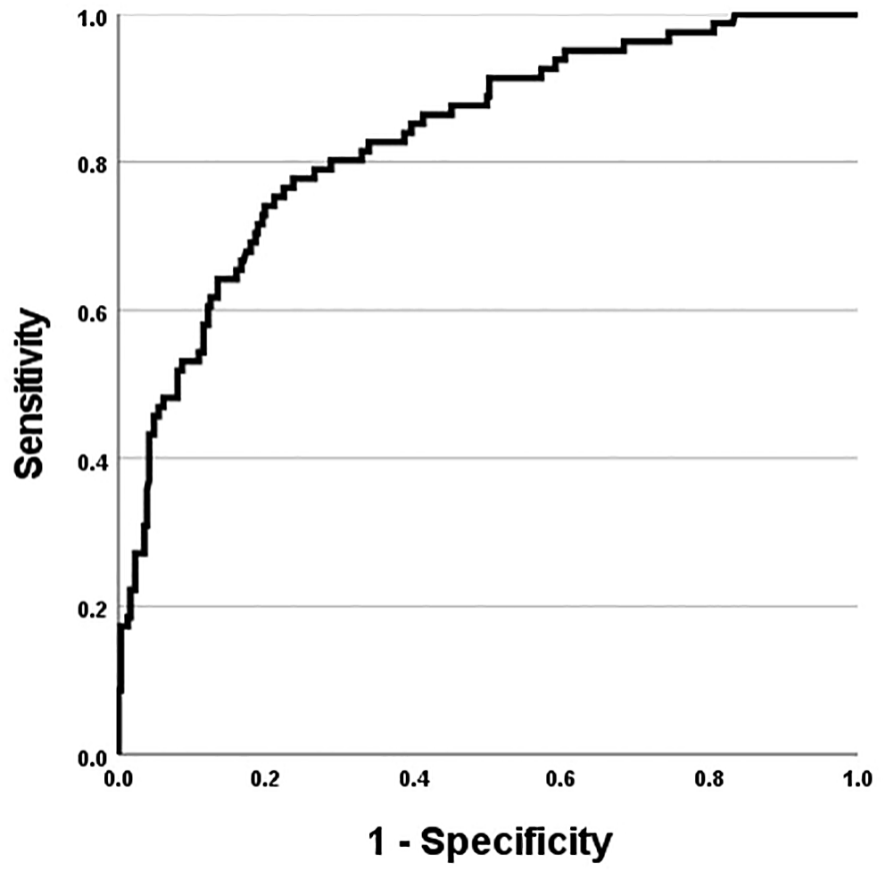
ROC curve for the final multivariable logistic regression model predicting significant self-harm (SHI≥25). with an area under the curve (AUC) of 0.832.

The ROC curve was used to evaluate the model’s discriminatory performance, yielding an area under the curve (AUC) of 0.832 (**Figure 1**). Using a probability threshold of 0.5, the model achieved a positive predictive value (PPV) of 69.8% and a negative predictive value (NPV) of 87.1%, indicating good classification performance. In addition, the full model including all candidate predictors demonstrated acceptable discrimination (AUC = 0.784) and is presented in **Appendix A, Figure A1**.

## Discussion

The purpose of this study was to investigate and understand the determinants that contribute to non-suicidal self-injury (NSSI) among university students in Jordan. Using a sample of 394 participants, we assessed the lifetime prevalence of the SHI’s 22 self-harm behaviors and its associations with psychological, experiential, and demographic factors. Our results showed a prevalence of 20.56% of significant self-harm (SHI ≥5) among the study participants. Furthermore, high proportion of students (63.2%) endorsed at least one SHI-defined self-harm behavior. These findings align with the results from a previous study conducted in Jordan (4) and approaching the upper range observed in international college populations (9). Hamdan et. al, estimated that > 60% of the university students in Jordan thought of harming self (4) while Hanania et. al, estimated 22.6% prevalence of lifetime NSSI among Jordanian adolescents (33). Globally, the prevalence of NSSI in youth increased in recent years (34). Global systematic reviews and meta-analyses reported adolescent NSSI prevalence ranges between 11.5% and 33.8% (34). Our results are consistent with the findings of study conducted among medical students in Pakistan (35) which demonstrated 60.9% self-reporting of at least one form of NSSI in medical undergraduates (35).

Psychological distress showed the strongest association with self-harm in our study. Students who reported self-injury exhibited significantly higher levels of depression and anxiety symptoms. Chi-square analysis confirmed a strong association between greater psychological distress and increased severity of self-harm. These findings are consistent with a large cross-national college surveys report that showed a substantially greater proportion of students with a history of NSSI who meet the criteria for at least one mental disorder compared with non–self-injuring peers (9). A prior Jordanian study similarly found that anxiety was closely tied to self-harm: over two-thirds of students had social anxiety, and social-anxiety symptoms were the only significant predictor of deliberate self-harm in regression analyses (4).

Self-injury serves as an emotion-regulation strategy that temporarily alleviates negative effects in individuals with high internal distress (36). Individuals with depressive symptoms often use self-injury to relieve psychological pain (37). Anxiety likewise often co-occurs with NSSI, possibly because anxious individuals also struggle with emotional dysregulation (37). Supporting this, an Iranian study found significantly higher Self-Harm Inventory (SHI) scores among Iranian students with clinical depression and anxiety (38). This convergence underscores the central role of depression and anxiety related processes in youth self-injury, and it aligns with our results and with prior evidence.

Based on the literature, NSSI behavior in young people is a way to relieve negative emotions (39). The emotional cascade model (ECM) (40)indicates that difficulties in emotion regulation (DER) may accumulate negative emotions and trigger or worsen NSSI and Suicide Attempts (SA) behaviors. ECM claims that DER affects young people’s coping and potentially exacerbates their negative emotions which might increase their NSSI and suicide risk by triggering emotional cascades (40). NSSI and SA might occur at the same time, and they are both risk factors for suicidal behaviors (41). The process model of emotion regulation proposed five strategies for DER that may be involved in NSSI. NSSI might be used to substitute more painful situations (42). Youth may also use NSSI to alter their social situation and shift their attention away from unpleasant thoughts. NSSI may switch cognitions about the self through self-punishment or downgrading the self from higher-order to lower-order awareness. NSSI can also modulate emotional responses through physiological changes in endogenous opioids or parasympathetic nervous system activation (42).

Adverse life experiences also differentiated the self-harm severity. Students who reported childhood adversity or a family history of psychological disorders were more likely to engage in NSSI, consistent with prior findings among university populations (4, 9). Such experiences may impair emotional development and foster maladaptive coping strategies. Furthermore, after controlling depression and anxiety, these associations remained significant, indicating that childhood adversity and family psychiatric history may represent independent risk factors for NSSI that are not fully explained by depressive symptoms. Literature about NSSI proposed that interpersonal dynamics play a role in NSSI (43). NSSI may be used to communicate stress or to gain support or to avoid social situations that are experienced as harmful. NSSI may also occur as a way of peer affiliation or bonding. NSSI provides an escape through transforming hostile feelings towards others back on oneself. Studies about the risk factors for NSSI reported interpersonal processes, including interpersonal trauma, conflict, social rejection, bullying, and perceived low social support (43, 44).

In contrast, problematic technology use appeared to play a less prominent role. Although smartphone addiction was examined as a potential behavioral factor, it did not emerge as a major determinant of self-harm in this study. This indicates that the connection between technology use and NSSI may be more complex and indirect, potentially mediated by psychological distress or sleep disturbance rather than representing an independent risk pathway. Previous research conducted in East Asia has reported stronger associations between smartphone overuse and self-injury (7, 45), suggesting that cultural and contextual factors may influence how technology-related behaviors interact with mental health.

Demographically, our sample showed no significant male-female gender gap in NSSI rates. However, in the adjusted analysis, female gender appeared to have a lower odds ratio of significant NSSI. This is consistent with the results of another study who found higher male NSSI among Jordanian adolescents (33).Years of study also mattered: second-year students showed the highest NSSI severity, whereas senior students (fifth or sixth year) had the lowest. No other demographics (GPA, income, major, age group, living situation) showed significant associations. Living status (with family, alone, or with friends) did not significantly affect NSSI, Overall, these findings imply that self-injury among students cut across subgroups, with the strongest vulnerabilities linked to psychological and stress factors rather than demographic characteristics.

### Implications for practice

This research reinforced that self-injury in university students is closely tied to internalizing distress: students with minimal anxiety or depression had the minimal risk of NSSI, whereas those with elevated distress levels were much more likely to report NSSI. The high levels of psychological distress reported among youth in this study and in previous research in Jordan (46), underscore the urgent need for strengthening campus mental health services. NSSI is multidimensional with emotional, compulsive, impulsive, and potentially addictive components (47)Thus, interventions should be specially tailored to stopping behavior and developing healthier emotion regulation skills, The clustering of NSSI with internal distress calls for integrated interventions. Context sensitive campus mental health programs should be installed to screen for self-harm as part of routine assessments. University counselors and clinicians should be aware that a student presenting with mood or anxiety symptoms may be at elevated risk for self-injury. Interventions that teach coping and emotion-regulation skills, such as Dialectical Behavior Therapy (DBT) skills training could serve the dual purpose of alleviating depression and anxiety while reducing self-harm (48). Involving family is crucial for protecting and supporting young people for positive emotional regulation. Finally, addressing the stigma towards NSSI through in campus mental health awareness campaigns can mitigate the barriers and encourage the youth to disclose and seek help.

### Limitations

This study addressed a worrying gap of knowledge about NSSI among university students in Jordan. Collecting the data through an online survey gave the participants comfort and freedom to report about this sensitive and stigmatized research topic. However, this research has several limitations that should be considered. Cross-sectional design prevents inferring causality (for example, whether depression leads to non-suicidal self-injury or vice versa). All the data were self-reported, which introduces the possibility of recall and social desirability biases. The use of convenience sampling and the higher percentage of participants from a single large public university may limit the generalizability of these findings to all university students in Jordan. Students who chose to participate may differ systematically from non-respondents in terms of psychological distress, help-seeking attitudes, or interest in mental health topics, introducing potential selection bias. Consequently, the observed prevalence and associations should be interpreted as reflective of this sample rather than as population-level estimates. Additionally, the inability to calculate a response rate further limits assessment of non-response bias.

Some methodological constraints were present in the statistical analyses. In examining group differences, Chi-square tests were used to assess associations between categorical variables and self-harm severity. However, for some multi-category variables, low expected cell counts violated Chi-square assumptions. In such cases, we applied alternative non-parametric methods such as the Kruskal–Wallis test, which limited direct comparability across subgroups. Similarly, the binary logistic regression employed a stepwise approach, which is useful for identifying the most statistically robust predictors but may have excluded theoretically important variables that did not reach significance in this sample. Sparse data in some categories may also have affected the stability and reliability of regression estimates.

Finally, our sample consisted largely of young undergraduates from Jordan, which may limit the generalizability of these findings to other age groups, cultures, or clinical populations. Future longitudinal and multi-site research would help clarify temporal relationships, for example, by determining whether increases in anxiety precedes self-harm and extending these findings to more diverse populations.

## Conclusion

In summary, this study revealed a high prevalence of self-harm behaviors as measured by the SHI, with psychological distress, particularly anxiety and depression, being the strongest factors associated with self-harm. Additionally, students who reported childhood trauma and family psychological disorders were found to be at increased risk. In contrast, technology appeared to play a lesser role, with only a weak positive correlation observed between smartphone addiction and self-harm severity. The high prevalence of NSSI and its strong link to psychological distress highlight the need for programs that screen for self-harm and promote coping strategies and emotion-regulation skills to support these students. Further qualitative studies should be conducted to investigate the etiology of self-harm and to develop effective strategies for early identification and intervention.

## Data Availability

The raw data supporting the conclusions of this article will be made available by the authors, without undue reservation.
