# Supplementary material for: Exploring the determinants of non-suicidal self-injury among university students in Jordan: a cross-sectional study
Source: Front Psychiatry. 2026 Jan 23;16:1751707. doi: 10.3389/fpsyt.2025.1751707 (PMC12878654; doi:10.3389/fpsyt.2025.1751707)

|  |  | Wald | Sig. | OR | 95% C.I.for OR | |
| --- | --- | --- | --- | --- | --- | --- |
|  |  |  |  |  | Lower | Upper |
| Step 1^a^ | PHQ-9 | 58.129 | <0.001 | 1.182 | 1.132 | 1.234 |
| Step 2^b^ | PHQ-9 | 53.593 | <0.002 | 1.178 | 1.128 | 1.231 |
|  | Does anyone in your family suffer from a psychological disorder? | 14.274 | <0.003 | 3.465 | 1.819 | 6.603 |
| Step 3^c^ | SAS-SV | 5.937 | 0.015 | 0.967 | 0.942 | 0.994 |
|  | PHQ-9 | 54.078 | <0.001 | 1.211 | 1.151 | 1.275 |
|  | Does anyone in your family suffer from a psychological disorder? | 13.691 | <0.002 | 3.433 | 1.786 | 6.598 |
| Step 4^d^ | SAS-SV | 5.753 | 0.016 | 0.967 | 0.942 | 0.994 |
|  | PHQ-9 | 48.26 | <0.001 | 1.202 | 1.141 | 1.266 |
|  | Any negative / traumatizing experience growing up | 4.555 | 0.033 | 1.991 | 1.058 | 3.748 |
|  | Does anyone in your family suffer from a psychological disorder? | 9.835 | 0.002 | 2.916 | 1.494 | 5.691 |
| Step 5^e^ | SAS-SV | 4.763 | 0.029 | 0.97 | 0.944 | 0.997 |
|  | PHQ-9 | 50.464 | <0.001 | 1.21 | 1.148 | 1.275 |
|  | Gender (reference: female) | 5.841 | 0.016 | 2.101 | 1.151 | 3.836 |
|  | Any negative / traumatizing experience growing up | 6.01 | 0.014 | 2.261 | 1.178 | 4.342 |
|  | Does anyone in your family suffer from a psychological disorder? | 9.049 | 0.003 | 2.806 | 1.433 | 5.496 |

Appendix A

A forward stepwise (Likelihood Ratio) logistic regression was conducted to identify predictors of significant non-suicidal self-injury. All candidate variables were initially eligible for entry. Variables were added sequentially according to the likelihood-ratio test (entry criterion p < 0.05), and the procedure terminated when no remaining predictors met the entry threshold. Table A1 summarizes the order of entry and the significance of each predictor at the step it entered.

Table A1: Order of entry and significance of each predictor.

A:Variable(s) entered on step 1: PHQ-9.

B:Variable(s) entered on step 2: Does anyone in your family suffer from a psychological disorder?

﻿﻿﻿C:Variable(s) entered on step 3: SAS-SV.

﻿﻿﻿D:Variable(s) entered on step 4: Any negative / traumatizing experience growing up?

E: Variable(s) entered on step 5: Gender

**Figure A1** presents the ROC curve for the logistic regression model that included **all initial candidate predictors** prior to stepwise selection. The area under the curve (AUC) was **0.784**, indicating acceptable discriminatory performance for distinguishing students with significant self-harm behaviour from those without it. This full-predictor model is shown for comparison with the stepwise-selected final model (AUC = 0.832), illustrating the improvement in classification accuracy after variable selection.


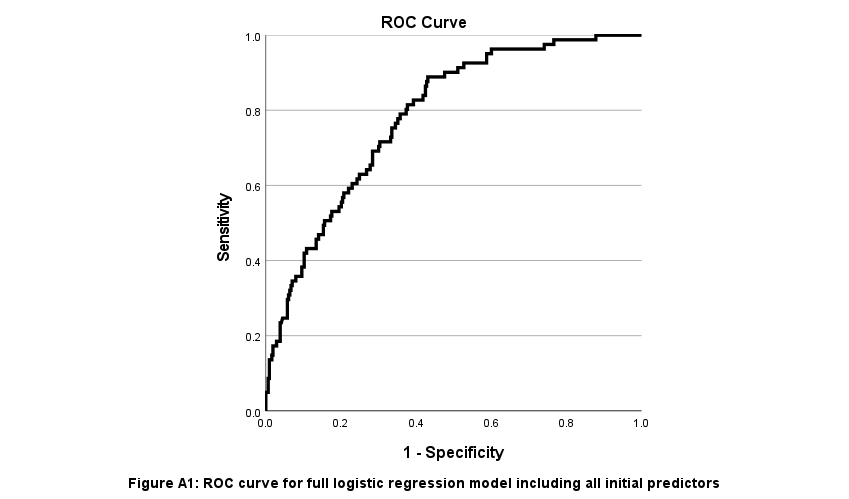

Supplement: Supplementary file 1 [file DataSheet1.docx]
